# Early detection of tuberculosis through community-based active case finding in Cambodia

**DOI:** 10.1186/1471-2458-12-469

**Published:** 2012-06-21

**Authors:** Mao Tan Eang, Peou Satha, Rajendra Prasad Yadav, Fukushi Morishita, Nobuyuki Nishikiori, Pieter van-Maaren, Catharina Lambregts-van Weezenbeek

**Affiliations:** 1 National Centre for Tuberculosis and Leprosy Control (CENAT), Ministry of Health, Phnom Penh, Cambodia; 2 World Health Organization, Representative Office in Cambodia, Phnom Penh, Cambodia; 3 World Health Organization, Regional Office for the Western Pacific, Manila, Philippines

## Abstract

**Background:**

Since 2005, Cambodia’s national tuberculosis programme has been conducting active case finding (ACF) with mobile radiography units, targeting household contacts of TB patients in poor and vulnerable communities in addition to routine passive case finding (PCF). This paper examines the differences in the demographic characteristics, smear grades, and treatment outcomes of pulmonary TB cases detected through both active and passive case finding to determine if ACF could contribute to early case finding, considering associated project costs for ACF.

**Methods:**

Demographic characteristics, smear grades, and treatment outcomes were compared between actively (n = 405) and passively (n = 602) detected patients by reviewing the existing programme records (including TB registers) of 2009 and 2010. Additional analyses were performed for PCF cases detected after the ACF sessions (n = 91).

**Results:**

The overall cost per case detected through ACF was US$ 108. The ACF approach detected patients from older populations (median age of 55 years) compared to PCF (median age of 48 years; p < 0.001). The percentage of smear-negative TB cases detected through ACF was significantly higher (71.4%) than that of PCF (40.5%). Among smear-positive patients, lower smear grades were observed in the ACF group compared to the PCF group (p = 0.002). A fairly low initial defaulter rate (21 patients, 5.2%) was observed in the ACF group. Once treatment was initiated, high treatment success rates were achieved with 96.4% in ACF and with 95.2% in PCF. After the ACF session, the smear grade of TB patients detected through routine PCF continued to be low, suggesting increased awareness and early case detection.

**Conclusions:**

The community-based ACF in Cambodia was found to be a cost-effective activity that is likely to have additional benefits such as contribution to early case finding and detection of patients from a vulnerable age group, possibly with an extended benefit for reducing secondary cases in the community. Further investigations are required to clarify the primary benefits of ACF in early and increased case detection and to assess its secondary impact on reducing on-going transmission.

## Background

Successful expansion of the internationally recommended directly observed treatment, short-course (DOTS) strategy since the mid-1990s has achieved remarkable progress in TB control in many parts of the world. The strategy, which promotes direct observation of therapy under one of its key principles, has contributed substantially to treatment success across the globe [1]. The World Health Organization (WHO) reported that 46 million people were successfully treated between 1995 and 2010, and the treatment success rate at the global level increased by 30% during this period [2]. Despite progress in treatment, however, case detection has stagnated in recent years [2,3].

The DOTS strategy promotes passive case finding (PCF), whereby patients with signs and symptoms of TB present themselves at health facilities for diagnosis and treatment [4]. While this conventional approach has been recognized as cost-effective [5], people who have limited access to TB services often fail to receive timely diagnosis and life-saving treatment. Delays in seeking care have been widely observed in various settings, particularly in TB high-risk and vulnerable populations, which has posed major challenges for improving case detection [5,6]. Active case finding (ACF), on the other hand, systematically looks for cases of TB, rather than waiting for people to develop symptoms and seek treatment. Although ACF has been implemented for decades primarily in resource rich settings, there is growing interest in using this approach for early case detection in developing countries [4,7].

Cambodia, one of 22 countries with a high burden of TB, has faced stagnation in case notification and has strived to increase case detection [2,8]. The country has adopted many case-finding approaches to promote early diagnosis, including ACF among contacts of TB cases at the community level [8]. Since 2005, the National Centre for Tuberculosis and Leprosy Control (CENAT), Cambodia, has been conducting outreach ACF sessions with mobile radiography units to target household contacts of TB patients in poor and vulnerable communities. Looking back in the history of TB control, ACF has been discouraged for many years because it is prohibitively expensive in some settings especially when it is conducted as a population screening and/or where TB prevalence is low [4]. Despite general recommendations against community-based ACF [9-11], these activities have been conducted effectively in Cambodia at a relatively low cost, employing an innovative approach that promotes retrospective contact investigations combined with symptom screening by community workers. However, the cost-effectiveness of the ACF approach and potential benefits for the patients, particularly comparing ACF with the conventional PCF approach, have yet to be examined.

This study examined whether ACF contributes to early detection of TB cases by comparing the smear grades of actively and passively detected pulmonary TB patients. The study also examined the potential benefits of ACF by analysing the demographic differences of patients and evaluated the cost-effectiveness of the ACF approach. The results of the study constitute an evidence base for future policy formulations on ACF in Cambodia.

## Methods

### Programmatic information

The ACF sessions were organized by CENAT in coordination with peripheral TB control staff and health services. Target operational districts (ODs) were selected based on the TB burden and vulnerability of the communities. About 7-10 days prior to the scheduled ACF session, local health workers visited community health volunteers and community leaders, and with them, visited household contacts of smear-positive patients who had registered for treatment in the past two years, and orally advised them to present at prearranged health centres on the day of the ACF session. If the immediate neighbours of the index cases had TB symptoms, productive cough lasting two weeks or more, they were also invited for the session. If the suspects were not at home, they continued to be followed up by community health volunteers. On the day of the ACF session, CENAT team together with local health staff set up a screening venue including mobile X-ray and microscopy stations. All TB suspects who presented to the ACF session underwent chest X-ray screening. The films were read by an experienced radiologist and categorized according to predefined criteria that included the categories of ‘normal’, ‘active TB’, ‘TB suspected’, ‘healed’, and ‘others’. Those in the categories of ‘TB suspected’, ‘healed’, and ‘others’ were further evaluated based on the individual risk of infection including clinical history to see if sputum-smear microscopy is required. For all in the ‘active’ category and those who required further investigations, three sputum samples were collected and examined by microscopy with Ziehl–Neelsen method on the day of the visit and on the following day in collaboration with a local laboratory. A senior physician on the ACF team diagnosed smear-negative TB based on radiological, clinical, and physical findings that were consistent with TB but not explained by other illnesses. Local health centres initiated treatment for both smear-positive and smear-negative patients.

Beside this distinctive ACF activity, TB cases registered under the routine programme were diagnosed according to national guidelines. Most cases were self-referral patients presenting to the health centres where a sputum smear microscopy is available. In case TB is still suspected in smear-negative subjects after anti-biotic trial, they should be referred to district health hospital for chest X-ray and subsequent assessment for diagnosis of smear-negative TB.

### Quantitative data

**Figure 1:**
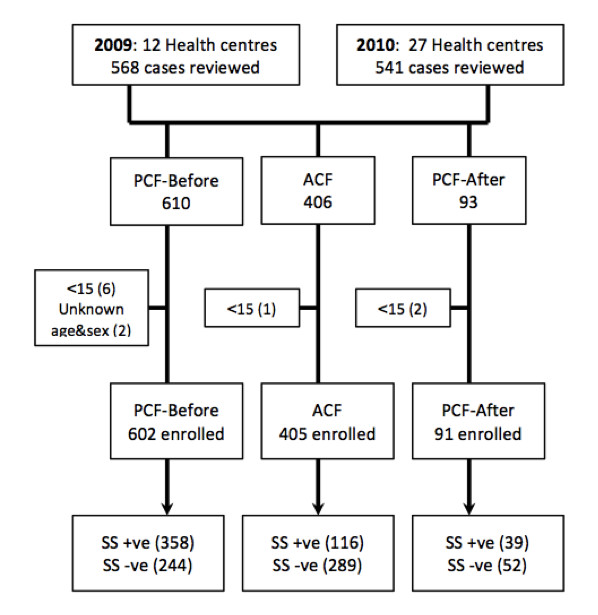
**Sampling process.** <15 = Patient is under 15 years of age. ACF, active case finding; PCF, passive case finding; SS + ve, sputum-smear positive; SS -ve, sputum-smear negative.

ACF sessions were conducted in 39 health centres across eight ODs in 2009 and 2010. We reviewed the records of all pulmonary TB cases detected by both active and passive case finding in the designated health centres (including PCF records from the same quarter and two quarters prior to the ACF sessions). Patients under 15 years and those with unknown sex and age were excluded from the study. As a result, out of 1109 TB case records that were reviewed, 405 ACF cases and 693 PCF cases were enrolled in the study (Figure 1). Among the PCF cases, 91 cases were registered after the ACF sessions and composed a separate group. Hence, the dataset consists of three groups: PCF-before, ACF, and PCF-after.

Primary variables such as smear grade and demographic characteristics were sourced from existing TB and laboratory resisters kept at CENAT. Smear grades were defined and recorded as follows: scanty (1–9 acid-fast bacilli [AFB] per 100 fields), 1+ (10–99 AFB per 100 fields), 2+ (1–10 AFB per field), and 3+ (>10 AFB per field) [12].

As this study used existing programme records and no personal identifying information was collected, ethical clearance was not required according to local regulations.

### Statistical analysis

The raw data were entered into a Microsoft Excel (Microsoft, USA) spreadsheet with predefined coding and error-checking formulas. The statistical analyses were performed using R 2.14.1 (CRAN: the Comprehensive R Archive Network at http://cran.r-project.org/). Distribution and frequency of smear grades, demographic characteristics, and other key variables were calculated and compared between the PCF-before and ACF groups. Pearson's chi-square test or Fisher’s exact test were applied to examine associations. In addition, to compare age distributions by case finding strategies, Mann-Whitney test was applied. The PCF-after group was used later as a secondary comparable group to assess the impact of ACF in the community.

## Results

### Number needed to screen

Between 2005 and 2010, 33 631 TB suspects who presented to the ACF sessions were registered for screening across Cambodia and all were screened by chest radiography. Among them, 5844 (17.4%) underwent sputum-smear microscopy, and 885 (2.6%) were found to be smear-positive patients. Hence, the number needed to screen (NNS) to identify one smear-positive case was 38.

### Cost of active case finding

Cost information was available only for ACF sessions conducted in five ODs in 2010. A total of 2561 suspects were screened by chest radiography, of which 337 (13.2%) underwent a sputum-smear test. These tests resulted in the detection of 56 smear-positive and 101 smear-negative cases (2.2% and 3.9% of all participants, respectively). Applying the local standard unit costs of US$ 1.20 for chest X-ray and US$ 0.70 for sputum-smear test per slide, the diagnostic cost per case detected including smear negatives was US$ 24 (ranging from US$ 16 to US$ 41 among five ODs). Using the overall project cost of US$ 16 917, including the costs of logistic support and human resources (with additional pay for the time involved in the activities but excluding basic salaries), the calculated overall cost per case detected was US$ 108.

### Demography

A comparison of the cases found by ACF and PCF-before is summarized in Table 1. Both case-finding methods detected slightly more female patients than male patients, which is consistent with overall case notification in Cambodia [2]. Although ACF yielded a slightly larger proportion of female patients compared to PCF, the difference was insignificant. 

**Table 1:** Characteristics of patients by case-finding method

	**ACF (%) (n = 405)**	**PCF-before (%) (n = 602)**	**P-value (chi-squared test)**
**Sex**			
Male	178 (44.0)	278 (46.2)	0.527
**Age group**			
15–24	10 (2.5)	44 (7.3)	<0.001
25–34	25 (6.2)	100 (16.6)	
35–44	51 (12.6)	104 (17.3)	
45–54	111 (27.4)	158 (26.2)	
55–64	114 (28.1)	108 (17.9)	
>65	94 (23.2)	88 (14.6)	
**Smear status**			
Negative	289 (71.4)	244 (40.5)	<0.001
Positive	116 (28.6)	358 (59.6)	
Smear grade of smear-positive cases**			
Scanty	10 (8.6)	8 (2.3)	0.003*
1+	56 (48.3)	143 (40.4)	
2+	30 (25.9)	137 (38.7)	
3+	20 (17.2)	66 (18.6)	
**Treatment initiation**			
Initial defaulter	21 (5.2)	NA	NA
**Patient category**			
New	384 (100)	592 (98.3)	0.006*
Re-treatment	0 (0)	1 (0.2)	
Transfer-in	0 (0)	3 (0.5)	
Others	0 (0)	6 (1.0)	
**Treatment outcome**			
Success (cure and complete)	370 (96.4)	573 (95.2)	0.323*
Default	3 (0.8)	4 (0.7)	
Died	3 (0.8)	11 (1.8)	
Transfer-out	0 (0.0)	4 (0.7)	
Failure	0 (0.0)	0 (0.0)	
Unknown/not evaluated	8 (2.1)	10 (1.7)	
**HIV status**			
Unknown	145 (35.8)	186 (30.9)	0.120
Known	260 (64.2)	416 (69.1)	
- Positive	1 (0.4)	4 (1.0)	0.696*

* Fisher’s exact test was employed due to small cell counts.

** Smear grades were not available for four cases in the PCF-before group.

**Figure 2:**
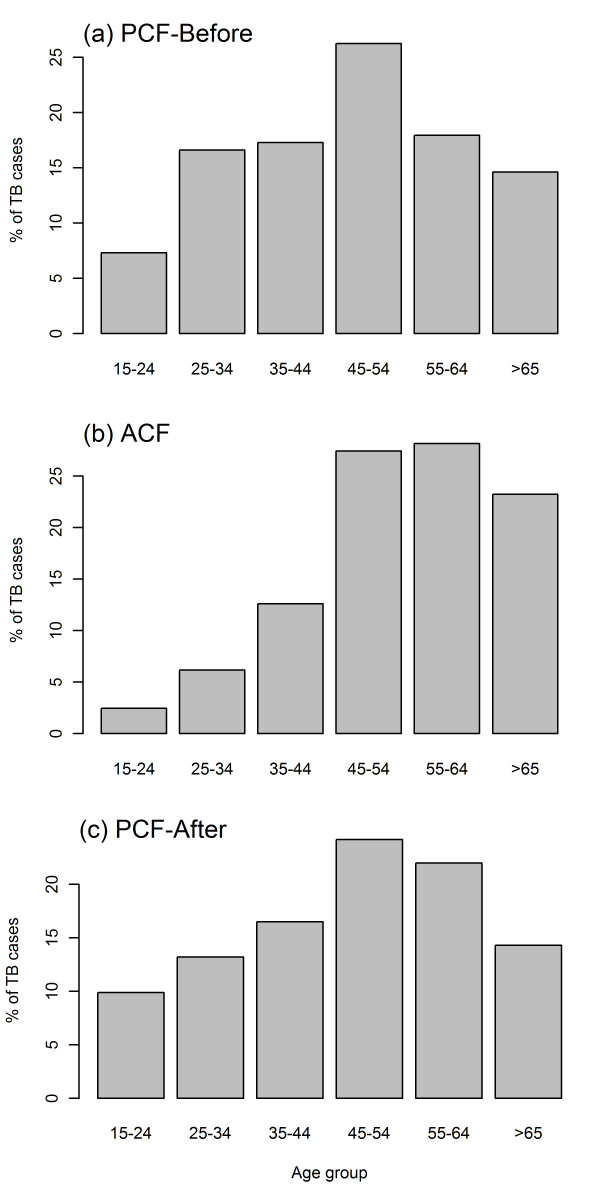
Age distribution of cases by case-finding method.

In terms of age distribution, ACF detected a higher proportion of older patients compared to PCF. The difference was statistically significant, with the median (IQR; inter-quartile range) being 55 (IQR 47-64) years in the ACF group and 48 (IQR 36-59) years in the PCF-before group (p < 0.001 by Mann-Whitney test). Figures 2(a) and 2(b) clearly indicate a shift in age distribution by case-finding method, with the highest proportion of cases found in the 45–54 year age group for PCF-before and the 55–64 year group for ACF. Patients aged 55 or older accounted for 32.5% in the PCF-Before group, while the ACF group had a higher proportion of 51.3%.

### Result of sputum-smear test

**Figure 3:**
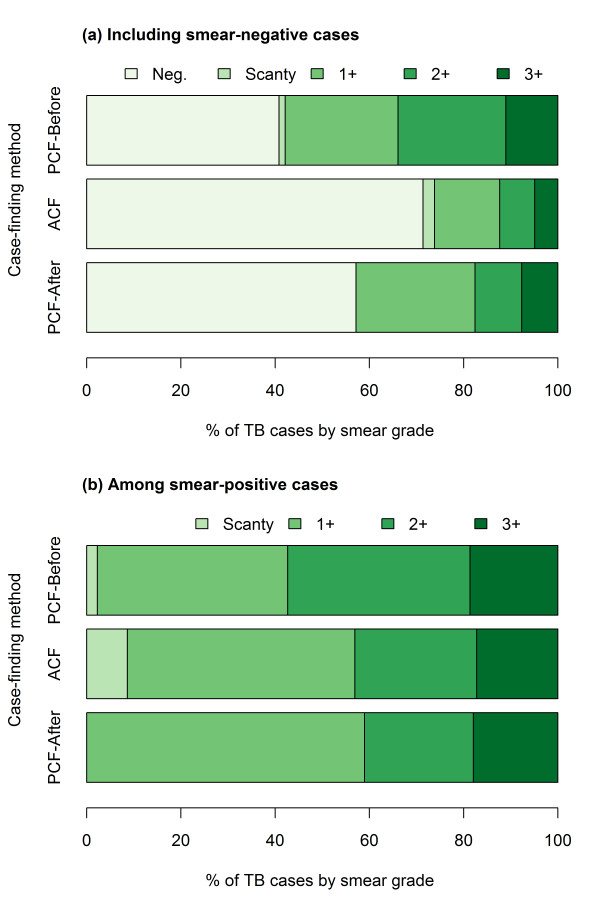
Distribution of smear grade by case-finding method.

The ACF group had higher proportions of smear-negative patients and smear-positive patients with lower smear grades than the PCF-before group. In fact, the percentage of smear-negative TB in the ACF group was significantly higher than that of the PCF-before group (71.4% and 40.5%, respectively, as shown in Figure 3(a); p < 0.001). Among the smear-positive patients, lower smear grades were observed in the ACF group compared to the PCF-before group, which was statistically significant (Figure 3(b); p = 0.002). Cases with lower smear grades (i.e. scanty and 1+) accounted for 56.9% of all smear-positive cases in the ACF group, compared to 42.7% in the PCF-before group (p < 0.010).

### Treatment outcome

In the ACF group, no TB suspects dropped out during the diagnostic pathway after the registration for ACF sessions. However, 21 patients (5.2%) including five smear-positive cases were not registered for treatment in the quarter of the ACF session or the following quarter. These patients are considered as initial defaulters who were lost to follow-up before treatment initiation. Among those registered, all actively detected patients were categorized as new cases, whereas other types of patients were also reported in PCF. Both case finding methods achieved a fairly high treatment success rate with more than 95%, excluding initial defaulters. The proportion of unfavourable outcomes (default, died, failure, and transfer-out) was slightly higher in the PCF-before group although this difference was not statistically significant. A small number of HIV-positive cases were reported from both case-finding methods.

### Patients detected through PCF after the ACF sessions (PCF-after)

Of the 91 cases detected through PCF after the ACF sessions, 45 (49.5%) were male and the median age was 49 (IQR 36.5-60) years, revealing similarities with the PCF-before group in respect of age distribution (Man-Whitney test results; p < 0.001 between ACF and PCF-after groups, while p = 0.541 between PCF-before and after groups). The smear status, however, was more like that of the ACF group, with 39 (42.9%) smear-positive cases (Figure 3[a]). The grade distribution was also similar to the ACF group, as shown in Figure 3(b). The number of cases diagnosed as 1+, 2+, and 3+ were 23 (59%), 9 (23.1%), and 7 (17.9%), respectively. Among them, one was HIV-positive, one was previously treated, and three died. The treatment success rate was 96.7%.

## Discussion

The community-based ACF approach employed in Cambodia, which focussed on TB contacts in poor communities with a high burden of TB, was found to be cost-effective, considering that the overall cost per case detected was US$ 108 and the NNS was 38. In comparison, eight FIDELIS projects (Fund for Innovative DOTS Expansion through Local Initiatives to Stop TB), supported by the Canadian International Development Agency (CIDA), reported a cost per case detected ranging from US$ 60 to US$ 1626 by ACF [13], which implies that the Cambodian ACF approach is comparably cost-effective. Besides, the NNS of 38 is very similar to those reported through intensified case finding in HIV-prevalent and congregate settings [14]. Considering the low HIV prevalence in this study population, the low NNS further justifies the cost-effectiveness of the strategy. The results of this study also revealed that the ACF approach could contribute to the detection of smear-negative TB cases or smear-positive TB cases with low smear grades. The approach could also detect more TB patients from older age groups, which demonstrates an “equalizing role” for ACF in TB service provision, especially as the elderly run the risk of dying without any attempt to diagnose the underlying source of their complaints or disease.

The elderly are generally considered vulnerable to TB as they belong to age cohorts with high TB infection rates, have relatively low immune status and limited access to health services [15,16]. A survey conducted in Cambodia in 2002 demonstrated that TB prevalence was up to seven times higher in the older age groups than in the general population. Yet the level of case detection among them was low [17]. In that sense, detecting TB among older patients could be seen as an additional value of ACF in the Cambodian context. Studies conducted in South India and Eastern Nepal showed similar results [9,18]. Some may argue that ACF in Cambodia missed out part of the younger population since most of the sessions were conducted during the day, which is more convenient for the elderly and not for the economically active age group. Yet one of the primary objectives of ACF is to identify and treat more patients who have remained undetected in the community with special attention to health inequity. Given that more than half of actively detected patients were older than 55 years of age compared to around one third of passively detected patients being older than 55 years of age, it is reasonable to conclude that the ACF approach has successfully complemented routine PCF in Cambodia by detecting older patients. The finding that all of the actively detected patients were new cases without any history of previous treatment further supports its complementary function.

Smear grade, a quantitative measure of TB bacilli in the sputum samples, has been suggested as a parameter that can represent the severity and the infectiousness of the disease [19,20]. The lower smear grades observed in the ACF group in this study, therefore, suggest that actively detected patients were at a relatively early stage of their disease. This was consistent with the findings of Santha et al. in South India [9]. Another study exploring the association between smear grade and diagnostic delay in Japan showed that patients with delayed diagnosis had significantly higher smear grades than those with timely diagnosis [21]. This finding implies that there is a link between smear positivity and timing of diagnosis, and further supports our argument that ACF can contribute to early case finding in a developing country setting. Looking at the newly detected cases among contacts of index smear-positive cases, Liippo et al. concluded that limiting contact investigation to close contacts of patients heavily positive by sputum smear makes contact tracing more effective [19]. From a different angle, Lin et al. examined the effect of treatment delay on secondary TB infection among household contacts [22]. The study demonstrated a linear positive relationship between the delays in treatment of index cases and the secondary attack rates among household contacts. Thus, both existing literature and our findings strongly suggest that ACF can reduce diagnostic delay and thereby reduce the risk of further transmission.

As programmatic operational research using routine programme data, our study has several limitations. It is important to note that smear grade is both directly and indirectly influenced by other factors including volume and quality of sputum specimens, capacity of laboratory technicians, and possibly characteristics of screened subjects such as HIV and smoking status [23-27]. However, as smear slides were read by local technicians under routine conditions within the standard external quality assurance system, these factors may not have been serious confounders. Nevertheless, it may be desirable to obtain additional evidence on ACF by conducting, for instance, a delay study (comparing the extent of various delays from onset of symptoms, treatment seeking, final diagnosis, to treatment initiation between ACF and PCF) to reconfirm our findings and further quantify its contribution to early case detection.

Another limitation may have been the diagnosis of smear-negative TB during the outreach sessions. Our findings, showing a larger proportion of smear-negative TB detected in ACF than in PCF, might have been partly due to the different diagnostic methods used in between ACF and PCF, and over-diagnosis of smear-negative TB during the ACF sessions. A similarly high proportion of smear-negative TB was reported in a study conducted in South India [9]. To overcome this limitation, CENAT has been implementing a new project employing Xpert MTB/RIF as more sensitive diagnostic tool.

Our data lack the information on the number of households visited and individuals interviewed in the communities which could serve as primary denominators of NNS. However, our results are still valuable by using the number of individuals attended in the ACF sessions as shown in the result.

Finally, this study was unable to perform a comparative analysis between ACF and PCF in terms of cost-effectiveness since cost calculation of PCF requires an extensive assessment of the health system costs. A proper cost-effectiveness analysis should also include the assessment of disability-adjusted life-years (DALY) lost under the conditions with or without ACF. Obviously, these are beyond the scope of this field-based operational research study.

As one of the negative aspects of ACF, the issue of initial defaulters has been discussed in several articles [4,9,18,28]. Refusal to start treatment might be attributed to a lack of motivation due to the absence or mildness of symptoms [9]. Although the available evidence is limited and its definition may vary depending on researchers, the initial defaulter rates in ACF reported in other countries ranged from 26% to 32% [9,28], which was much higher than our observation of 5.2%. Taking into account that the TB programme in Cambodia has maintained a high treatment success rate and a low defaulter rate at national level over the years [2], these programmatic strengths may be reflected in the low initial defaulter rate among actively detected cases. This assumption suggests that ACF is likely to result in more initial defaulters in places with poor TB programmatic indicators, and further suggests that, when adopting ACF, such indicators need consideration in order to maximize effectiveness of the activities.

In this study, the smear grades of patients diagnosed through PCF after the ACF sessions were as low as those of actively detected patients, which might imply that ACF had a sustained impact on smear positivity among patients in the community. The one-time ACF sessions identified and treated many patients with relatively mild symptoms as well as those with heavy bacterial load. This may have contributed to the reduction of the overall patient pool in the community and thus led to the lower smear grades among patients in routine PCF. In addition, taking into account that the previous national prevalence survey was conducted in 2002, those reviewed in the study had not been exposed to massive ACF or TB awareness campaigns for a substantial period of time. Thus our ACF activities were likely to have contributed to increased community awareness about the disease and availability of services. Community mobilization might have promoted early health seeking and diagnosis.

## Conclusion

Cambodia’s ACF approach targeting TB contacts in communities was found to be a cost-effective approach that is likely to have additional benefits such as contribution to early case detection and detection of patients from a vulnerable age group, possibly with an extended benefit for reducing secondary cases in the community. Further investigations are required to clarify the primary benefits of ACF in early case detection and to assess its secondary impact on reducing on-going transmission.

## Competing interests

The authors declare that they have no competing interests.

## Authors’ contributions

NN and RPY designed the study, developed the research protocol and supervised data collection. FM and PS substantially contributed to the acquisition and interpretation of the data. NN and FM performed statistical analyses and drafted the first version of the manuscript. MTE, RPY, PVM, and CL have been involved in revising it critically for important intellectual content. All authors read and approved the final manuscript.

## Pre-publication history

The pre-publication history for this paper can be accessed here:

http://www.biomedcentral.com/1471-2458/12/469/prepub
